# Scaling and universality in urban economic diversification

**DOI:** 10.1098/rsif.2015.0937

**Published:** 2016-01

**Authors:** Hyejin Youn, Luís M. A. Bettencourt, José Lobo, Deborah Strumsky, Horacio Samaniego, Geoffrey B. West

**Affiliations:** 1 Institute for New Economic Thinking, University of Oxford, Oxford OX2 6ED, UK; 2 Mathematical Institute, University of Oxford, Oxford OX2 6GG, UK; 3 Santa Fe Institute, 1399 Hyde Park Road, Santa Fe, NM 87501, USA; 4 The Center for Social Dynamics and Complexity, Arizona State University, Tempe, AZ 85281, USA; 5 School of Sustainability, Arizona State University, Tempe, AZ 85281, USA; 6 Santa Fe Institute Center for Biosocial Complex Systems, Arizona State University, Tempe, AZ 85281, USA; 7 Instituto de Conservación, Biodiversidad y Territorio, Universidad Austral de Chile, Valdivia, Chile; 8 Instituto de Ecología y Biodiversidad, Casilla 653, Santiago, Chile; 9 Mathematics Department, Imperial College, London SW7 2AZ, UK

**Keywords:** universality, power laws, urban indicators, scaling laws, urban scaling

## Abstract

Understanding cities is central to addressing major global challenges from climate change to economic resilience. Although increasingly perceived as fundamental socio-economic units, the detailed fabric of urban economic activities is only recently accessible to comprehensive analyses with the availability of large datasets. Here, we study abundances of business categories across US metropolitan statistical areas, and provide a framework for measuring the intrinsic diversity of economic activities that transcends scales of the classification scheme. A universal structure common to all cities is revealed, manifesting self-similarity in internal economic structure as well as aggregated metrics (GDP, patents, crime). We present a simple mathematical derivation of the universality, and provide a model, together with its economic implications of open-ended diversity created by urbanization, for understanding the observed empirical distribution. Given the universal distribution, scaling analyses for individual business categories enable us to determine their relative abundances as a function of city size. These results shed light on the processes of economic differentiation with scale, suggesting a general structure for the growth of national economies as integrated urban systems.

## 1. Introduction

Diversity is a defining characteristic of complex adaptive systems whether ecosystems, social systems or economies [1–4]. In particular, it has been argued that the success and resilience of cities, together with their role in innovation and wealth creation, are driven by their ever-expanding diversity [3–8]. The presence and ever-changing admixture of individuals, ethnicities, cultural activities, businesses, services and social interactions is a defining characteristic of urban life. Together with its counterpart, specialization, this is often cited as to what makes a city unique and distinctive, and has consequently featured prominently in the study of cities across economics, geography and urban planning [9]. Despite its acknowledged importance, however, there have been surprisingly few quantitative investigations into possible systematic regularities and underlying dynamics that govern the diversity of cities across an entire urban system. A recurrent goal in developing an overarching science of cities is to discover, and conceptually understand, general patterns for how people, infrastructure and economic activity are organized and inter-related [10–13]. Compelling questions therefore are: how is diversity related to aggregate urban socio-economic and infrastructural metrics, and how do these quantities depend on city size?

The systematic quantitative understanding of diversity raises several conceptual issues that need to be addressed. For example, measuring diversity typically involves identifying different (business) types and counting their frequency for a given unit of analysis, such as a city or a nation [1]. It should be immediately clear that such a task can be problematic because any systematic classification scheme is subject to an arbitrary recognition of specific categories, as any business type can be further subdivided as long as a defining distinction is made. Restaurants, for example, can be decomposed into fine dining, fast food, etc., as well as into multiple levels of cuisine, price, quality, etc., so, in general, urban diversity is scale-dependent [14]. Firstly, the challenge, therefore, is to seek a resolution-independent characterization. Secondly, we need to deconvolute the intricate relations between scale, diversity and economic productivity, as well as between diversification and specialization. To be sustainable, successful new business types must lead to higher economic productivity by introducing, for example, greater specialization and interdependence that involve larger numbers of people, both as workers and clients. Thus, we might expect that the larger scale of bigger cities, and hence higher productivities, should afford greater economic diversity (at least in absolute terms) though such an expectation is potentially at odds with the idea that it is specialization that drives increase in efficiency.

In this paper, we measure and characterize economic diversity in order to clarify its underlying role in economic development. Our analysis reveals a surprising systematic behaviour common to all cities. We show how this can be derived theoretically and present a simple model for understanding its structure based on a variant of preferential attachment for introducing new business types. The model quantitatively predicts how individual business types systematically change rank with city size, shedding light on processes of innovation and economic differentiation with scale.

We focus on the frequency distribution of business types (the number of ‘species’) and first ask how this varies across cities (the ‘ecosystem’). We identify our unit of analysis as the *establishment*, which is defined as a single physical location where business is conducted, so that, for example, individual stores of a national chain would be counted separately. Establishments are nowadays seen as fundamental units of economic analysis because innovation, wealth generation, entrepreneurship and job creation all manifest themselves through the formation and growth of workplaces [15]. We explore a unique dataset, the National Establishment Time-Series, a longitudinal database built by Walls and Associates, to capture economic life at an extraordinarily fine-grained level [16,17]. This dataset includes records of nearly the entire set of establishments (work places) in US urban areas (over 20 million) each of which is classified according to the North American Industry Classification System (NAICS) in 2008. We aggregate such information into the standard definition of functional cities: the 366 metropolitan statistical areas (MSAs), which are defined by the census bureau as unified labour markets centred on a single large city wielding substantial influence over its surrounding region [18]. These MSAs account for over 90% of the economic output of the US and encompass almost 85% of its population (the lower limit on MSA size is approx. 50 000).

## 2. Results

**Figure 1.**
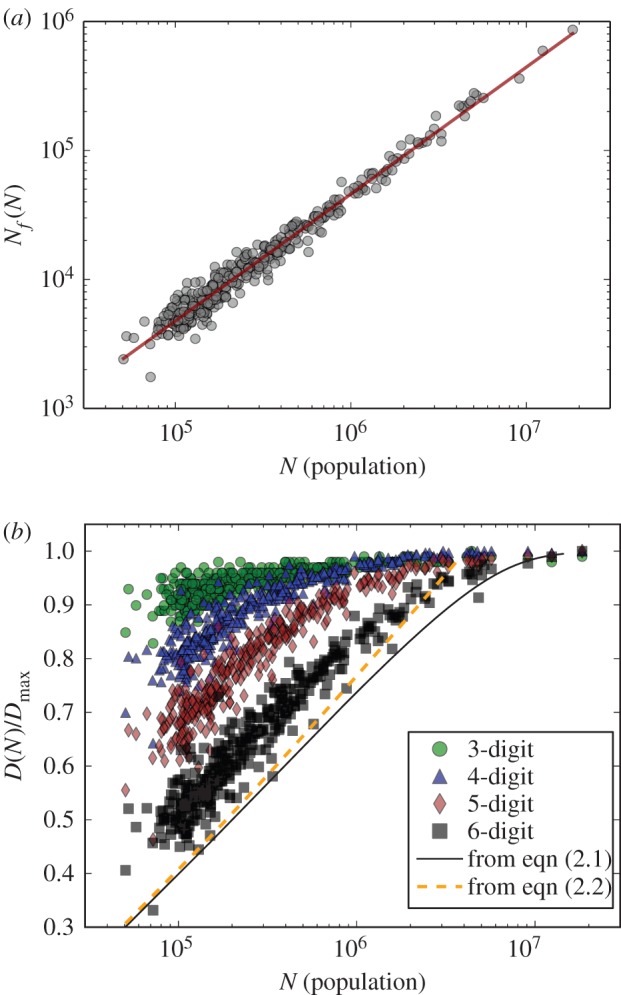
(*a*) The total number of establishments *N_f_* scales linearly with city size: *N_f_* ∼ *N^*α*^*, where *α* = 0.98 ± 0.02 with *R*^2^ = 0.97. (*b*) The number of distinct business types *D*(*N*) normalized by its maximum value *D*_max_ at various levels of classification, *r*, based on the NAICS scheme, from the lowest resolution (three-digit) to the highest (six-digit) denoted by green circles, blue triangles, red diamonds and black squares, respectively (corresponding values of *D*_max_ are 317, 722 and 1160). All values are scaled by the corresponding size of the classification scheme at that resolution, *D*_max_, such that all values fall in between 0 and 1. The black solid line and orange dashes are the predictions from equation (2.5), with and without *ϕ*.

The data reveal a surprisingly simple result. The total number of establishments, *N_f_*, in each MSA is linearly proportional to its population size, *N*: that is,2.1
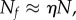
with the proportionality constant 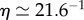 (figure 1*a*). Thus, there is approximately one establishment for every 22 people in a city, *regardless* of its size; or, to put it slightly differently, on average a new work place is created each time the city size increases by 22 people. Combined with the fact that the number of employees, *N*_e_, also scales approximately linearly with *N*, 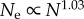, the average size of establishments is also independent of population size; that is, the number of employees per establishment, 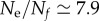 is independent of *N*. The remarkable constancy of the average number of employees and the average number of establishments across cities is not only contrary to previous wisdom [19,20], but also somewhat puzzling when viewed in light of agglomeration effects on establishment size with *per capita* increases in productivity, wages, GDP or patent production, with population size [21–23], corroborating the external economies of scale [24,25]. Therefore, the economic composition of cities deserves closer scrutiny.

The simplest measure of economic diversity counts distinct establishment types, *D*(*N*), within a metropolitan area of population *N* [1,26]. Figure 1*b* shows *D*(*N*) as a function of *N* at successive resolution levels as defined by the NAICS dataset. At all resolutions, *D*(*N*) increases logarithmically with *N*, but eventually levels off. This saturation, observed in a number of different datasets [14,27,28], has been attributed to the limit where the finite NAICS classification scheme in the largest cities cannot fully capture the true extent of economic diversity: when means of differentiation among work places is insufficient, saturation is inevitable. For example, the category *restaurants* in NAICS could be further deconstructed into different cuisines: Thai, Indonesia, Korean, Japanese, Chinese, etc. This effect can be traced out by using the hierarchical structure of NAICS; see the electronic supplementary material text for the detailed methods. Figure 1*b* (coloured circles) shows that *D* is indeed not only a function of *N* but also a function of the resolution of the classification scheme, *r*. As the resolution becomes finer the logarithmic increase (the orange dotted line in figure 1*b*) remains intact while the saturation to its maximum value, *D*_max_(*r*), appears at larger and larger city sizes. This suggests that saturation is, in fact, an artefact of insufficient detail in the description of business types. We return to this point below where the logarithmic functional form of *D*(*N*) will be derived from the universal scaling distribution of business types to which we now turn.

**Figure 2.**
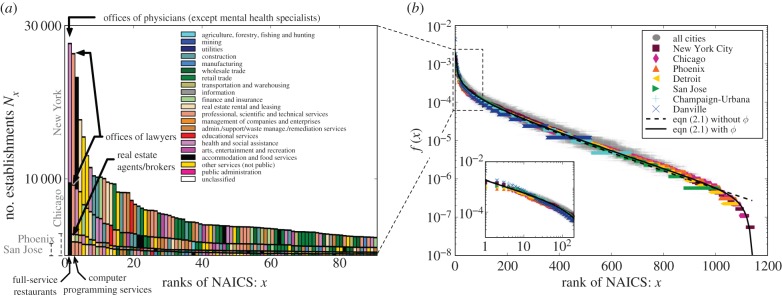
Rank-abundance of establishment types. (*a*) The number of establishments at rank *x* ranging from 1 to 90 in descending order of their frequencies (from common to rare) for New York City, Chicago, Phoenix and San Jose. Establishment types are colour coded by their classification at the two-digit level. (*b*) Universal rank-abundance shape of the establishment type by dividing *N_x_* by the population size of city in semi-log for all ranges. All 366 metropolitan statistical areas are denoted by grey circles. Seven selected cities are denoted by various colours and shapes; New York city, Chicago, Phoenix, Detroit, San Jose, Champaign-Urbana and Danville are, respectively, marked by red squares, pink diamonds, orange triangles, yellow left triangles, green right triangles, sky blue pluses and blue crosses. The black dash line and the black solid line are fits to equation (2.3) without and with *ϕ*, respectively. The inset shows the first 200 types on a log–log plot showing an approximate Zipf-like power law behaviour.

A more insightful way of assessing economic diversity is to examine the constituent types of *D*(*N*) for individual cities. The abundance of the 100 leading business types for a selection of cities is shown in figure 2*a*; see the electronic supplementary material for more examples and further rank abundance figures. In New York, the most abundant business type is *offices of physicians*, followed by *offices of lawyers* and *restaurants*; Phoenix ranks *restaurants* first and *real estate* second (perhaps, not surprising in a rapidly growing city); San Jose, which includes Silicon Valley, predictably ranks *computer programming* second only to *restaurants*. Indeed, the composition of economic activities in cities has its own distinctive characteristics reflecting the individuality of each city. It is, therefore, all the more remarkable that, *despite the unique admixture of business types for cities, the shape of these distributions is universal; so much so that, with a simple scale transformation, their rank abundances collapse to a single unique curve common to all cities* (figure 2*b*). Note that the curve is robust to changes in levels of income, density and population size, which vary widely across the USA; see, the electronic supplementary material.

This universality can be derived from a sum rule for the total number of establishments as follows. Let *F_i_*(*N*) be the number of the *i*th most abundant business type in a city of size *N*, as shown in figure 2*a*. When summed over all ranks, this must add up to the total number of establishments, *N_f_* (*N*): thus, 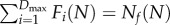. But, from equation (2.1), *N_f_* (*N*) = *ηN*, so introducing the *per capita* distribution, 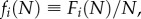 this can be re-expressed as2.2
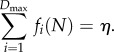
As *N* increases, any growing or diverging dependences of the *f_i_*(*N*) on *N* in equation (2.2) cannot be cancelled against each other as they are all positive definite, while any decreasing dependence vanishes. As *η* is a constant this implies that each *f_i_* must itself become independent of *N* for sufficiently large *N*, predicting that the *per capita* frequency of a given rank *i* must be the same for all cities. Note that the derivation is independent of the detailed underlying dynamics. Figure 2*b* verifies the predicted invariance of *f_i_* across all cities. The derivation of this invariance remains valid when we treat the discrete rank, *i*, as a continuous variable, *x*, and correspondingly *f_i_* as a continuous function, *f*(*x*); see the electronic supplementary material. The surprise in the data is that this predicted collapse to a single curve extends all the way down to relatively small cities mirroring a similar precocious scaling observed in urban metrics as a function of population.

The universal form of this scaled rank-size distribution, *f*(*x*), has three distinct regimes: for small *x* (<*x*_0_, say), it is well described by a Zipfian power law with exponent *γ*, as shown in the inset of figure 2*b*; for larger *x*(>*x*_0_), it is approximately exponential; and finally, as *x* approaches its maximally allowed value for the total number of categories at a given resolution, *D*_max_, *f*(*x*) drops off precipitously. These can be combined into a single analytic form:2.3
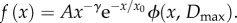


Figure 2 shows a fit to the data with the parameters *γ* ≈ 0.49 and *x*_0_ ≈ 211 both independent of *N* and of the cut-off function, *ϕ*(*x*, *D*_max_), to a good approximation. The overall normalization, *A*, is not an independent parameter but is determined from the sum rule for *f*(*x*), equation (2.2), which gives *A* ≈ *f*(1) ≈ 0.0019 in excellent agreement with the data.

The function, *ϕ*(*x*, *D*_max_), parametrizes the cut-off in *x* that is enforced by the finite resolution of the classification scheme. To a large extent, it is determined by imposing three general conditions that it must satisfy:

(i) *ϕ*(*x*, *D*_max_) = 1 when 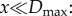 this expresses the constraint that the cut-off is only important when *x* is close to *D*_max_.
(ii) *ϕ*(*x*, *D*_max_) → 1 when *D*_max_ → ∞: its effect vanishes when the resolution becomes sufficiently fine.
(iii) *ϕ*(*D*_max_, *D*_max_) = 0: the cut-off completely dominates when *x* reaches its endpoint at its maximum value determined by the finite resolution, *x* = *D*_max_.

A simple phenomenological function that satisfies all of these conditions is2.4
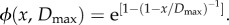
See, the electronic supplementary material for possible generalizations that include an additional parameter. For comparison, we show fits to the data both with and without *ϕ* in figures 1*b* and 2*b* to illustrate the effect of *ϕ*, which only become important when *x* approaches its maximum value.

Recall that *D*_max_(*r*) is the maximum possible number of business categories that can appear in a city using a given classification scheme with resolution *r*, and so consequently increases with increasing resolution; see the electronic supplementary material. In the limit of the finest grained resolution, *r* → ∞, we expect *D*_max_ → ∞, in which case the term *ϕ* in equation (2.4) becomes constant, turning off the saturation phenomenon; this is point (ii) above. This effect is already visible in figure 1*b*, where saturation sets in at smaller city sizes for coarser resolution. This leads to the possibility that the highest level of current resolution of the NAICS classification scheme may be insufficient to capture the actual business type diversity of the very largest cities.

The cut-off, *ϕ*, is, therefore, attributed to the saturation observed in figure 1*b*. This can be also shown by deriving the diversity function *D*(*N*) from the rank-size distribution *f*(*x*) (including the cut-off *ϕ*). This construction is possible because *D*(*N*) corresponds to the lowest possible rank, *x*, containing only a single establishment. In the continuum limit this can be expressed as *F*[*D*(*N*)] = 1, or *f*[*D*(*N*)] = 1/*N*, leading to2.5
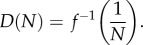
Given the form of *f*(*x*) in equation (2.3), this cannot be solved analytically except in certain limits. Instead, a numerical solution gives excellent agreement with data as shown in figure 1*b* (the black solid line). When the classification resolution *r* is sufficiently fine, *D*_max_ becomes large and *ϕ* becomes 1. In that case, this equation can be approximated:2.6
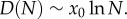
This result agrees with the numerical solution which, as anticipated, shows no saturation in *D*(*N*) (the orange dashed curve in figure 1*b*) This represents an open-ended ever-expanding diversity with population growth and confirms that the cut-off, *ϕ*, is associated with the saturation observed in *D*(*N*).

We can understand the structure of *f*(*x*) and *D*(*N*) in the context of generalized preferential attachment or growing models [29,30]. These models are a widely accepted mechanism for generating rank-size distributions, whether for words, genes or cities. They are based on a stochastic growth process in which new elements of the system (business types in this case) are attributed a probability, *α*, of creating a new type, or adding to an existing type [31,32]. In the classic Simon–Yule model, for example, the attachment probability, *α*, of being an existing type is proportional to the existing frequency. As a result, the model exhibits a feedback mechanism in which more-frequent types acquire new elements with higher probability than less-frequent types. Such a model provides a plausible mechanism for the observed Zipfian behaviour in the high frequency regime of *f*(*x*): namely, a power-law distribution *x*^−*γ*^ with *γ* = (1 − *α*) < 1 when *x* < *x*_0_ as in figure 2*b*. As *γ* in equation (2.3) is estimated from empirical data as 0.49, *α* is 0.51. This number suggests quite a rapid expansion so that small cities go through a stage of significant increase in business diversity as they grow: on average, about half of the new establishments entering the system spawn a new business type. This fast pace of diversification is in marked contrast to the pace in the exponential regime of *f*(*x*) where *x* is larger than *x*_0_. The Yule–Simon model can still be used to explain this exponential regime by relaxing the condition for *α*: the probability is no longer constant but a function of the city size *N*. Figure 1*b* shows that the probability of each business being a new type, *α*, decreases with city size. Because *α*(*N*) = d*D*(*N*)/d*N*, the logarithmic functional form of *D*(*N*) in equation (2.6) gives *α*(*N*) ∼ 1/*N*. This variation in *α*(*N*) can now be used in the model of aggregate growth to predict the exponent 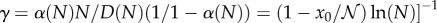, which vanishes for large *N*, and results in the observed exponential behaviour in *f*(*x*); see the electronic supplementary material for details.

The empirical findings (figures 1 and 2) coupled with the predictions of the model described above suggest that all cities, as they grow, exhibit the same underlying dynamics in the development of their business ecology. Initially, small cities, with a limited portfolio of economic activities, need to create new functionalities at a fast pace. These basic activities constitute the economic core of every city, big and small. Later, as cities grow, the pace at which new functionalities are introduced slows down dramatically, but never completely ceases. Large cities, then, presumably rely primarily on combinatorial processes for developing new relationships among their many existing functionalities, which in turn is the source of observed increases in economic productivity. This is a general feature of the combinatorial growth process, which has empirically been observed in international trade and US patent data: once the set of individual building blocks is large enough, their combination is sufficient to generate novelty even when the set itself expands slowly or not at all [33–35].

Clearly, the universal distribution of frequencies of business types does not account for the entire developmental process of economic functionalities in cities. The stochastic Simon–Yule model, for example, cannot predict what business compositions sit in what ranks and in what cities. If, during growth, the introduction and success of each establishment were independent of business type (but dependent on frequencies), there would be no statistical structure in how ranks are occupied. This is in clear disagreement with the observation shown below as well as with the pattern that ‘creative’ and innovative activities concentrate disproportionally in large cities [7,8,22]. Nevertheless, we can make some interesting and potentially powerful predictions about rank evolution of specific types as individual cities grow in size.

**Figure 3.**
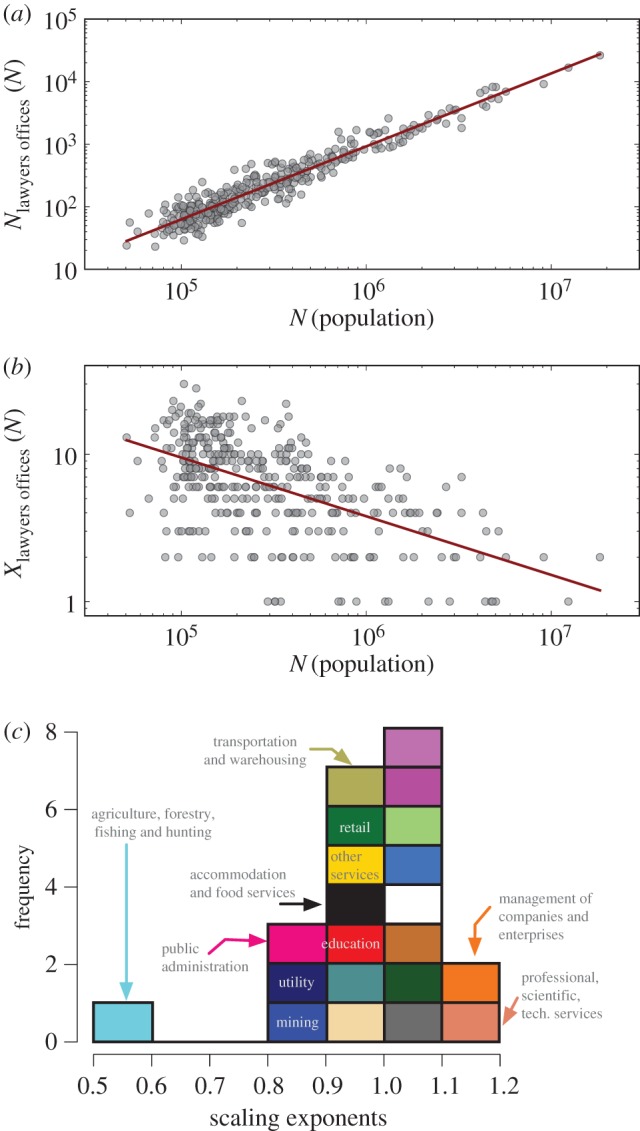
Multidimensional allometric scaling of industry types. (*a*) The number of lawyers' offices scales super-linearly with population size, *N*_lo_ ∼ *N*^1.17±0.04^ with *R*^2^ = 0.92. (*b*) The rank of lawyers' offices goes up with population size, expressing an increase in their relative abundance: *x*_lo_ ∼ *N*^−0.4±0.06^ with *R*^2^ = 0.32. (*c*) Histogram of scaling exponents *γ* for all establishment types at the two-digit level. While primary sectors disappear, managerial, professional, technical and scientific establishments increase in relative abundance, helping to explain the increased productivity of larger cities despite the slow addition of new business types. Each scaling analysis is shown in the electronic supplementary material.

The process by which specific business types assume different ranks in different cities may be particular to the ecology of specific places; or it may also be a property of scale. To distinguish between these two cases, we perform a multidimensional allometric scaling analysis of the number of specific establishments in each type. The super (or sub)-linearity of specific business types represents a systematic *per capita* increase (or decrease) of their abundances with city size. Figure 3*a* shows an example: the number of *lawyers' offices*, *N*_lo_, scales as *N*_lo_ ∼ *N^*β*^*, with *β* ≈ 1.17. That the exponent *β* is greater than one (super-linear) means that larger cities systematically have more lawyers' offices *per capita*. Because lawyers' offices typically appear at high frequencies (*x* < *x*_0_) we can approximate *f*(*x*) in equation (2.3) by its power-law behaviour and write, 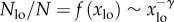 and thereby derive how the rank of lawyers' offices changes with city size: 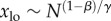. This predicts 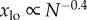, which is in good agreement with the actual scaling as shown in figure 3*b*. We can similarly predict how the ranks of low abundance business types scale. The rank shift can be expressed as:2.7
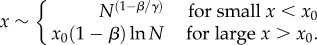

Thus, business types whose abundances scale super-linearly with population size systematically increase their rankings, whereas those that are sub-linear systematically decrease, as expected. This prediction works because the universal distribution holds.

Figure 3*c* summarizes the values of scaling exponents for business sectors at the two-digit level (see the electronic supplementary material for the histogram of exponents at the six-digit level). Most primary sectors such as, *agriculture*, *mining* and *utility* scale sub-linearly, predicting their systematic suppression, in relative terms, as cities get larger. On the other hand, informational and service businesses, such as *professional, scientific, and technical services* and *management of companies and enterprises* scale super-linearly, and are therefore predicted to increase disproportionally with city size, as observed. There are also sectors such as *restaurants*, arguably one of the most prominent and important examples of a city's non-tradable consumer goods, for example, that do not change ranks. Sectors that deviate from linearity tend to be tradable industries that may be exchanged across cities [8,36]. Because markets for these industries are not restricted to their immediate spatial location, comparative advantages may generate agglomeration economies resulting from city size and/or to specific places.

## 3. Discussion

The positive relationship between product varieties and city size has long been discussed in regional science, geography, economics and archaeology [5,8,9,14,21,24,25,27,28,37]. Many stylized facts identified by conventional approaches are, nevertheless, still subject to inconsistent granularity of categorization, so much so that existing empirical work tends to focus on particular industries and what is the appropriate classification scheme is left unclear. We address this issue by integrating the effect of classification resolution, the detailed statistics within individual cities and a stochastic model that explains the universal distribution. We conclude that recurring saturation in the largest cities is attributed to the finite resolution of the classification scheme that fails to capture the full extent of economic diversity [14,27,28]. In other words, the saturation in largest cities is inevitable when means of differentiation among work places is insufficient. Only when the finite-resolution effect is rectified, does the true diversity relation reveal itself an open-ended ever-expanding diversification process as population grows.

The capacity to generate open-ended diversity is one of the most important characteristics of many complex systems, from ecosystems to modern human societies. Cities, holding the majority of the world's population, are perhaps the best platform for studying the open-ended diversity in economies, as they form defined socio-economic units spanning over a broad range of scales. Furthermore, increasing economic diversity together with the specialization and division of labour is key to understanding the greater productivity and wealth of cities, and, eventually, to explaining the phenomenon of urbanization [8,9,21].

The distribution of business types in US cities is characterized by a universal rank-size curve in which specific types predictably increase or decrease their relative rankings and frequencies as a function of city size. Our model provides a good description of how statistics of business composition may be a result of a general mechanism of business creation based on existing frequencies—which yield observed historical paths—and how the rate of introduction of new types slows down with city size. This mechanism bespeaks an open-ended logarithmic increase in diversity. Nevertheless, we would like to point out that the model has much room for improvement in the future: it does not sit in a canonical economic framework, as it were, where equilibrium is assumed and optimal size of a city for an industry is derived [8,37–39] and its implications for growth dynamics are inferred from cross-sectional data due to the availability [40].

Clearly, much work remains to be done to create a more a complete picture. Nevertheless, the intuition gained from this model still demonstrates its merit in the following sense. First, the present model and scaling analysis provide an alternative angle that supports and complements the hypothesis advanced by classic central place and locational theories of cities [41,42] where a general hierarchy of economic activities is assumed. These classic observations need to be interpreted not only in terms of the appearance of specific new sectors with city size, but also with the *disproportionate* growth of *certain* types versus others. In our framework, scaling exponents capturing the disproportionate growth of sectors in size can parameterize such hierarchy order in a more systematic way than simple counts of businesses or [subjective] expert judgements on the nature of sectors, the approaches of which impose *a priori* categories of industry stages. Our alternative metric of hierarchy order is expected to be further developed to capture the *economic complexity index* that has recently been proposed in development economics [34,35]. Second, our work gives quantitative support to business life-cycle theories [43,44], where some types of business may be more prevalent in larger cities, but over time tend to move down the urban hierarchy as they mature and internalize more of their business model. This approach requires that we look at cities as a whole, in terms of urban systems, and not as isolated individuals. By looking at all cities and the complete set of business sectors that make up the entire urban economy in a country, our analysis links the economic fabric of each city to that of a system of cities. Finally, our model shares an interesting common feature of combinatorial innovation with the existing literature: small cities, with a limited portfolio of economic activities, acquire new functionalities at a fast pace to make up a core economy, then slow down, but never completely cease to innovate. This dynamic feature is consistent with the recent observation on the US patent data [33]. The basic functionalities existing in the economic core are combined to become innovative functionalities within firms, some of which, presumably, create new types of industrial activities that will further trickle down to smaller cities, and that will possibly be the source of observed increases in economic productivity [45]. We believe that the present results, together with further analyses of revenue, employment, temporal patterns, provide the foundation for more mechanistic understanding of how large cities realize greater economic productivity and how urbanization tends to promote nationwide economic growth.

## 4. Material and methods

The National Establishment Time-Series is a proprietary longitudinal database built by Walls and Associates [31,46]. This dataset includes records of nearly the entire set of *establishments* in US urban areas (over 20 million) each of which is classified according to the NAICS. ‘Establishment’ is a technical term to define a single physical location where the business is conducted (work place). A ‘firm’ or a ‘company’, on the other hand, is a legal business, either corporate or otherwise, and may consist of one, a few, or even a very large number of establishments [47–49]. The term establishment is used to highlight the importance of a single physical location of an organization and engagement in one or predominantly one activity as unit of economic activities [15]. Therefore, one city can have many Starbucks establishments, for example. We aggregate these establishments of various NAICS categories within a city. We use MSAs (v. 2008) as the functional city for our unit of analysis [18].

## Supplementary Material

Supplementary Material
