# Supplementary material for: Scaling and universality in urban economic diversification
Source: J R Soc Interface. 2016 Jan;13(114):20150937. doi: 10.1098/rsif.2015.0937 (PMC4759798; doi:10.1098/rsif.2015.0937)
Supplement: Supplementary Material [file rsif20150937supp1.pdf]

Supplementary Material

H. Youn, L. M. A. Bettencourt, J. Lobo, D. Strumsky, H. Samaniego, and G. West

Contents

1 Establishments data and methods **2**

2 Rank-size abundance distribution **3**

2.1 Rank-size distributions for selected cities 3

2.2 Effect of income level on deviations of the universal distribution 9

2.3 Mathematical derivation of the universality of the distribution 10

2.4 The universal shape 11

2.5 Growing model to explain the universal shape 12

3 Scaling analysis and rank shifts **14**

1 Establishments data and methods

Numbers of establishments by type and metropolitan statistical area were compiled from the National Establishment Time-Series (NETS) Database, which includes more than 20 million establishments in US cities 2008. An establishment refers to a single physical location where business is conducted, and is considered to be a fundamental unit of economic analysis [1]. Each establishment type in the dataset is classified according to the North American Industry Classification System (NAICS). We aggregate them into the US-Census-defined metropolitan statistical areas (MSAs). MSAs describe 366 unified labor markets with population scaling from 50 thousand people to almost 20 million (New York).

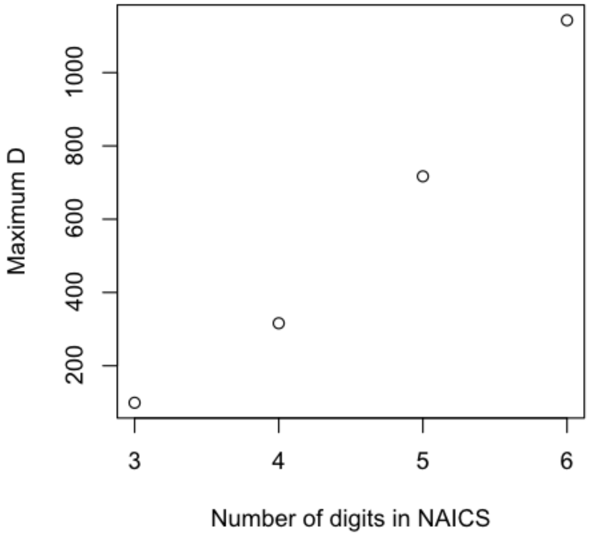

Figure 1: The number of distinct types of businesses in the largest city, $D_{\max}$, with different resolution schemes r in NAICS.

NAICS is the standard system for classifying industry and business establishments used by Federal statistical agencies [2]. It is a two- through six-digit hierarchical classification system, offering five levels of detail. Each digit in the code is part of a series of progressively narrower categories, and the more digits in the code signify greater classification detail. The first two digits designate the economic sector, the third digit designates the subsector, the fourth digit designates the industry group, the fifth digit designates the NAICS industry, and the sixth digit designates the national industry [2]. A complete and valid NAICS code contains six digits. For example, NAICS code 72, for example, denotes "accommodation and food services" which can be further divided into "food services and drinking places" (NAICS code 722), "limited-service eating places" (7222), "Cafeterias" (722212) and so on.

We parameterize the resolution of scheme with the number of digit r. As the resolution becomes finer, that is, larger r, the number of distinct types of business $D_{\max}(r)$ becomes larger, as shown in Fig. S1, and, correspondingly, saturation come in larger cities Fig. 1B. Most of analysis for $F_i(N)$ and f_i in the main text uses the data at the highest resolution $r = 6$. In the section 2.3, we explain that universal distribution holds in the high resolution.

2 Rank-size abundance distribution

In ecology, species abundance is a key element of biodiversity. It refers to how common or rare a species is relative to other species in a given community, where species potentially or actually compete for similar resources. Here, we consider establishment types as species and their number as their abundance in an ecosystem. Then, the species area relation corresponds to $D(N)$ where population N acts like area and D acts like the number of species as it is shown in Fig. 1B (main text); and universal rank-size distribution is f_i as shown in Fig. 2.

2.1 Rank-size distributions for selected cities

Here we display more examples of the rank-ordered frequency distribution of business types for several selected metropolitan areas: all MSAs (Fig. S2), New York (Fig. S3), Phoenix (Fig. S4), San Jose (Fig. S5), and Detroit (Fig. S6). Each figure shows the top and the bottom hundred rank business. It is interesting to note the persistent patterns that restaurants (black) and lawyers offices (orange) are always at high ranks (within the top ten) while manufacturings, minings, and utilities (expressed as blue shades) are at low ranks. The regularity of these rank positions across cities is derived by utilizing scaling and universality of the rank-size frequency distribution in the section S3.

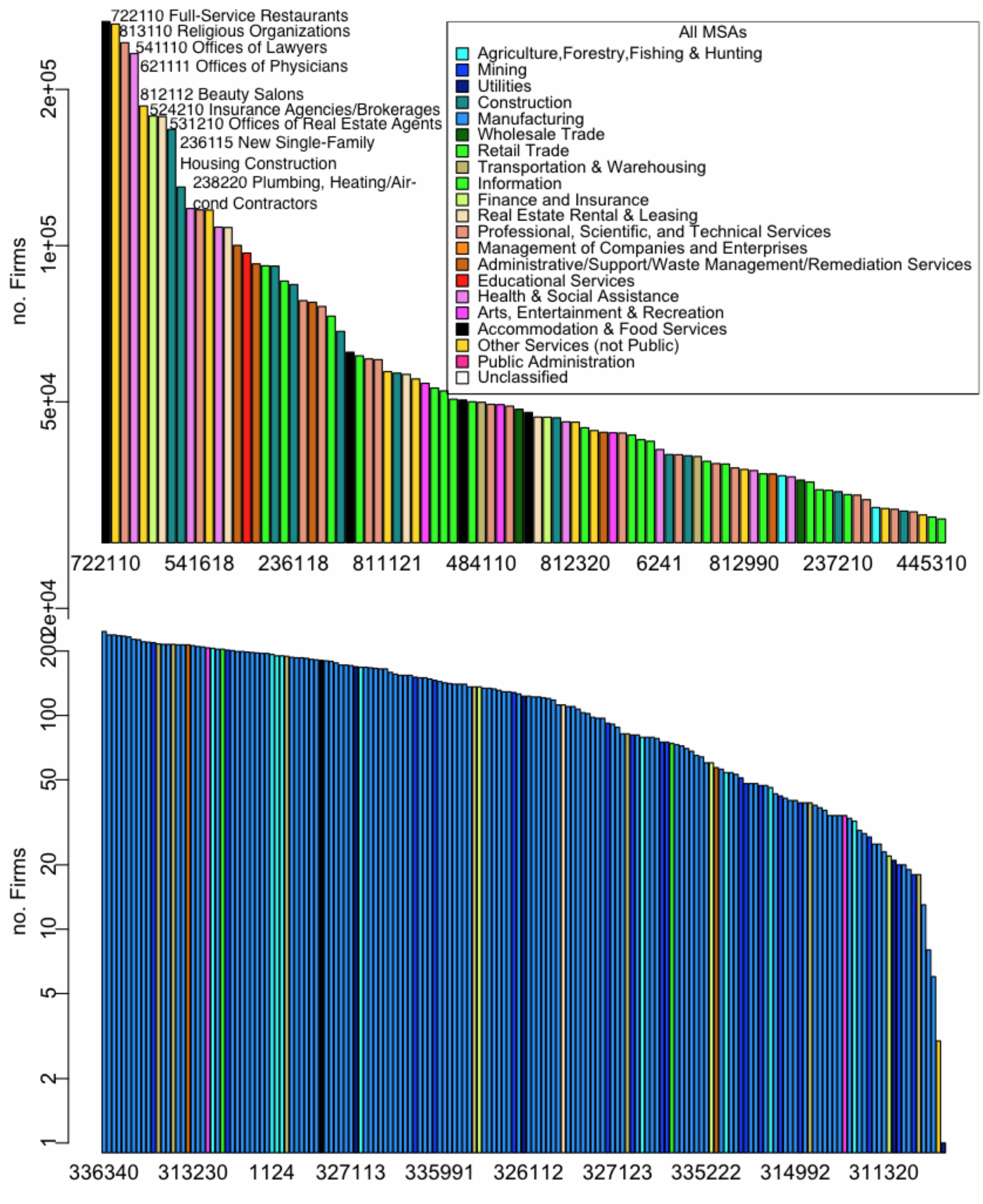

Figure 2: The top and the bottom hundred of the number of establishments in their descending order for all MSAs, $F_i(N)$, where i is rank. Types of establishments are color coded at their two digit correspondences.

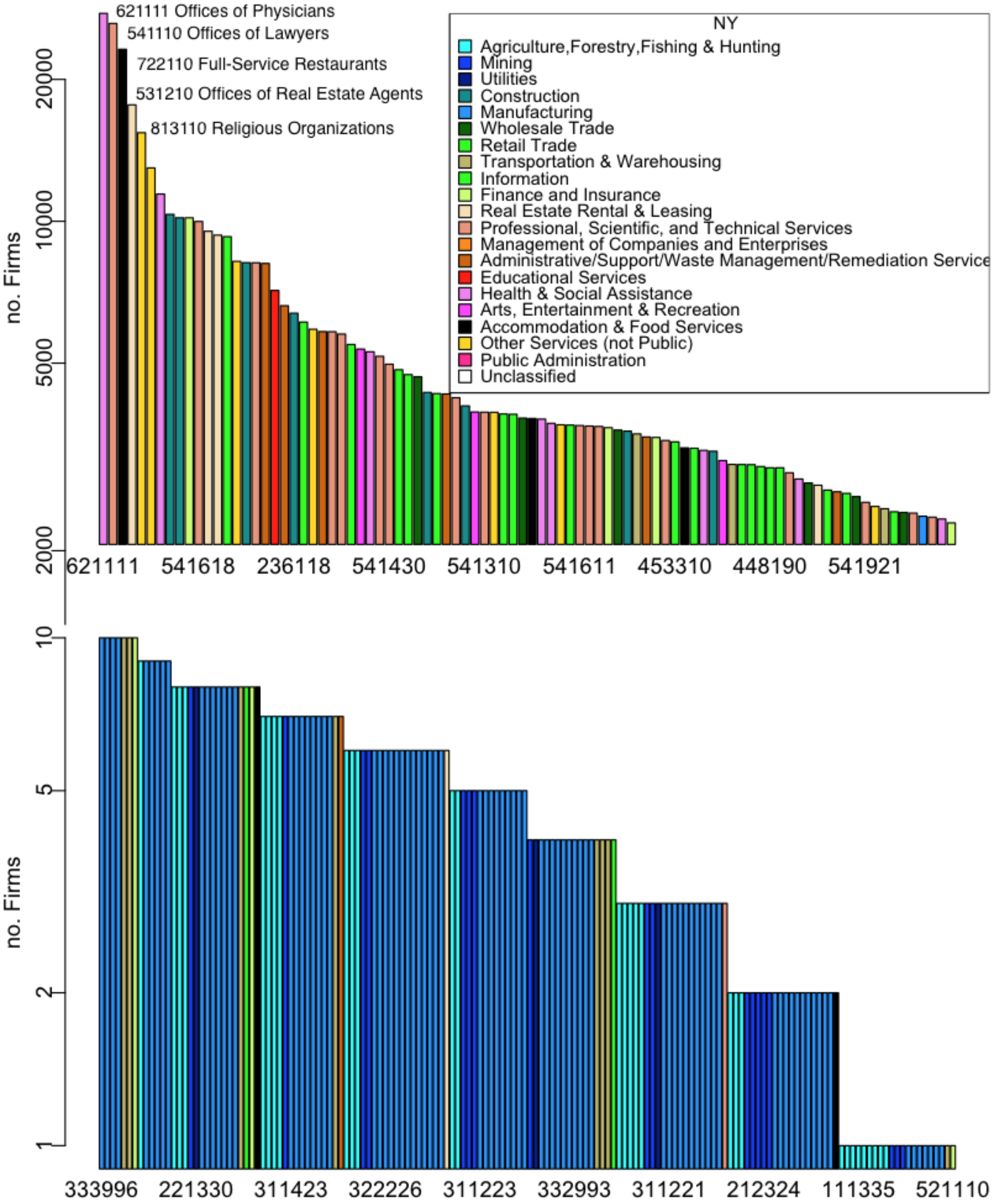

Figure 3: The top and the bottom hundred of the number of establishments in their descending order for New York City, $F_i(N)$, where i is rank. Types of establishments are color coded at their two digit correspondences.

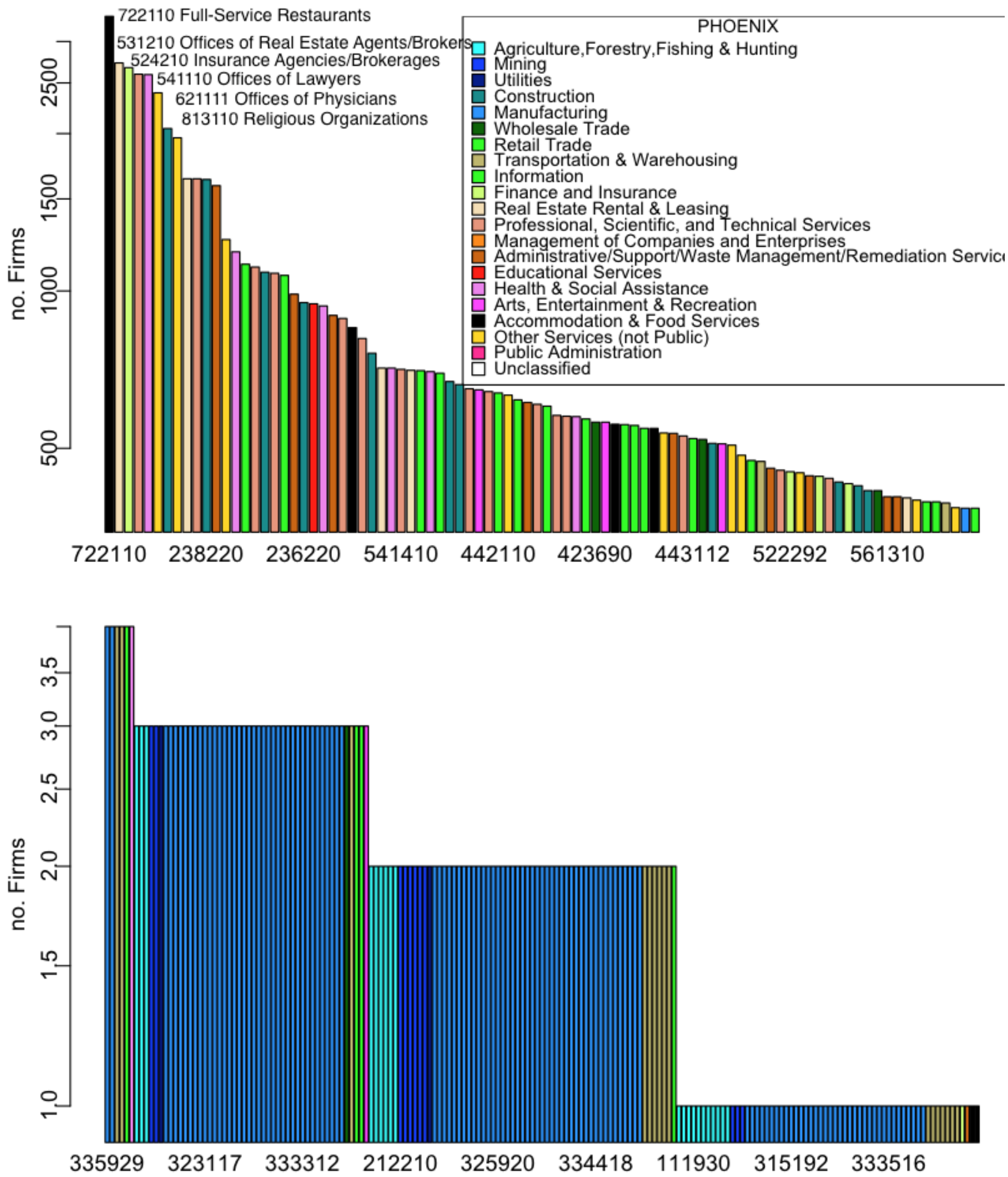

Figure 4: The top and the bottom hundred of the number of establishments in their descending order for Phoenix, $F_i(N)$. Types of establishments are color coded at their two digit correspondences.

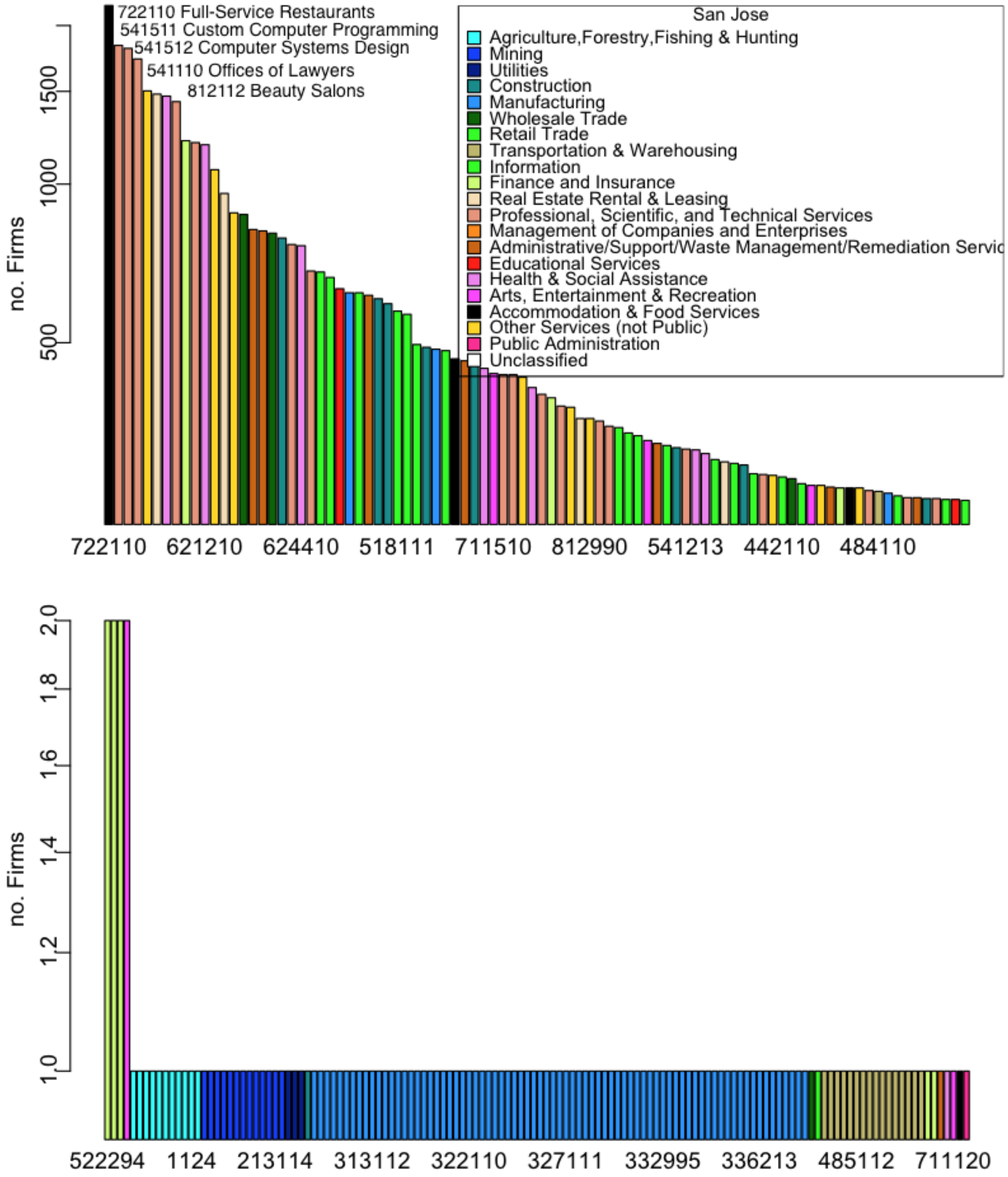

Figure 5: The top and the bottom hundred of the number of establishments in their descending order for San Jose, $F_i(N)$. Types of establishments are color coded at their two digit correspondences.

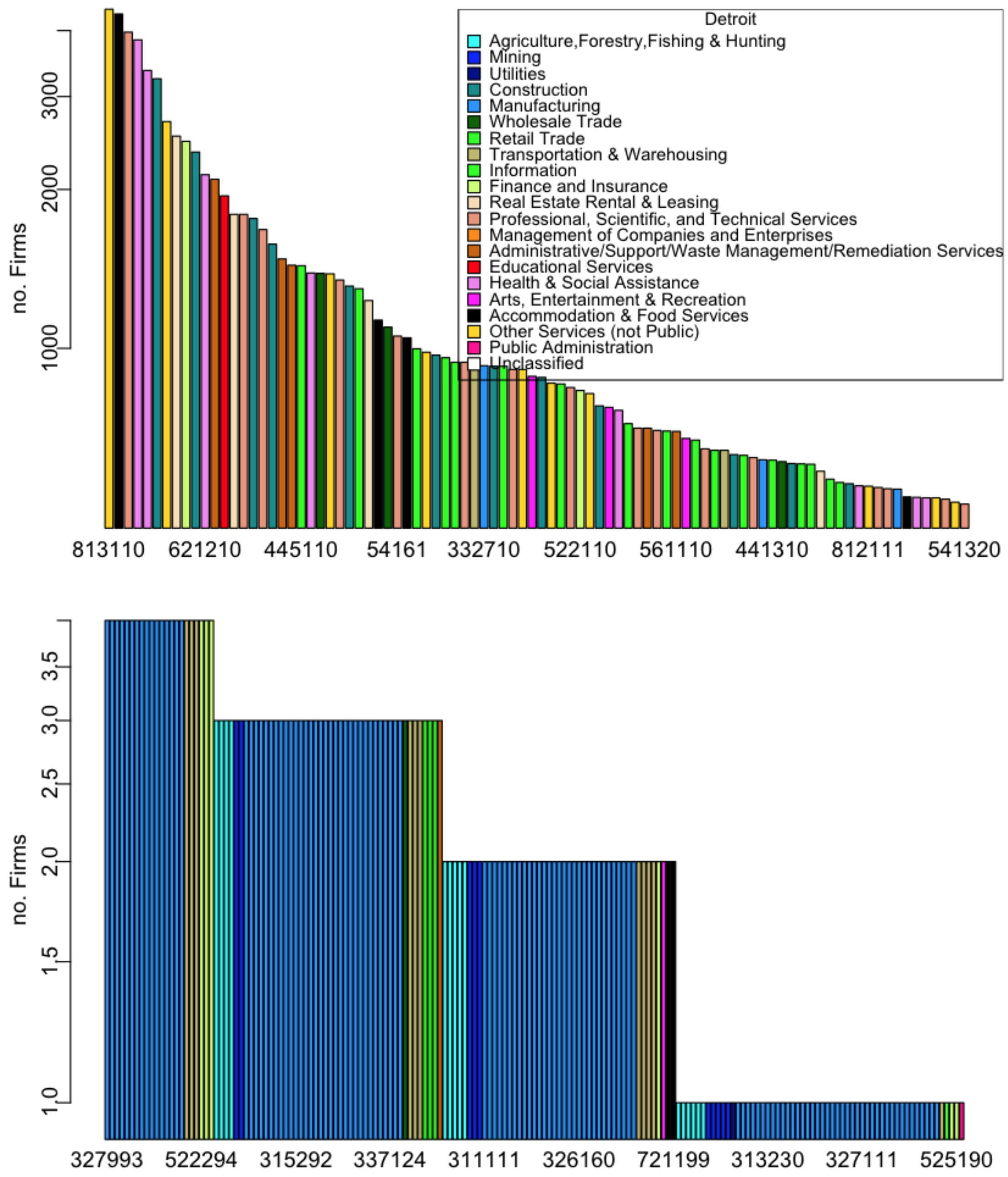

Figure 6: The top and the bottom hundred of the number of establishments in their descending order for Detroit, $F_i(N)$. Types of establishments are color coded at their two digit correspondences.

2.2 Effect of income level on deviations of the universal distribution

We study the effect of income level of cities on their shape of distribution. If citizens have higher income, business sectors may follow different dynamics that generates different distribution, which, then, may explain the deviations from the expected universal curve. Do cities of low-income or high-income level have different distribution of business sectors? We compare cities of various level of income such as Stamford, Brownsville, San Jose, El Paso and McAllen along with cities shown in the main text: New York, Chicago, Phoenix, Detroit and Santa Fe. The figure 7 shows that the cities of low-income level generally under-represent $F_i(N)$ than the cities of high-income level given the population size. This creates vertical down shift from the expectation attributing to deviation from the universal shape. However the shifts, and hence the deviations, do not mean that distribution shapes are deviated from the universal shape. This is shown in Fig. 7 (B) where $f(x)$ is rescaled by optimized population N' such that deviations are minimized. The deviation significantly disappears and the universal shape becomes even clearer and tighter.

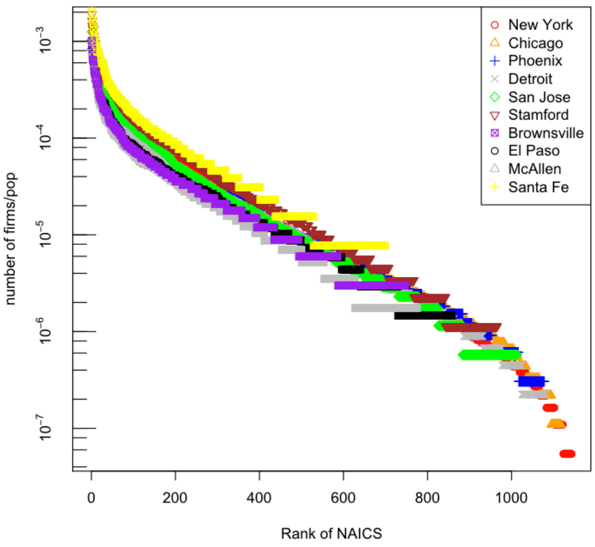

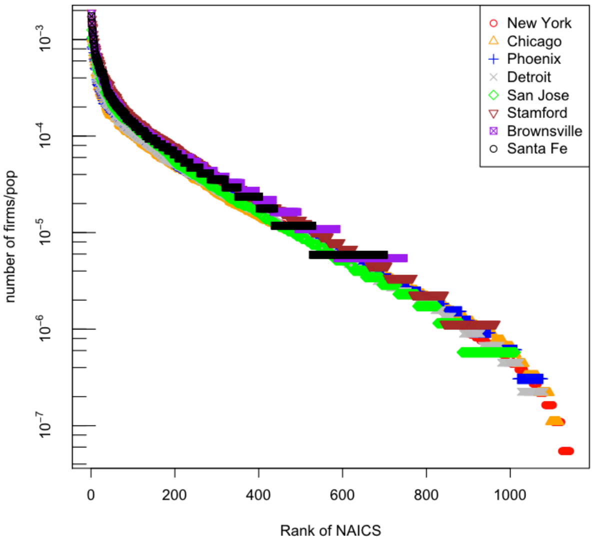

Figure 7: The empirical data with actual population scaling and with adjusted scaling by which we can observe that the shape is universal although their amplitudes of border cities (Brownsville, El Paso and McAllen) are different, and that the deviation from the universal shape is partly due to denominator. Cities are marked by the red circle, orange triangle, blue plus sign, gray cross, green diamond, brown down triangle, purple box, black circle, gray triangle and yellow plus sign for New York, Chicago, Phoenix, Detroit, San Jose, Stamford, Brownsville, El Paso, McAllen and Santa Fe, respectively. The second panel rescales the first panel to be in the tight comparison.

2.3 Mathematical derivation of the universality of the distribution

Although a universal function for the diversity of businesses across all cities seems counter-intuitive, the result can be heuristically derived in the limit of large cities using a mathematical argument based on a simple sum rule for the total number of establishments as below.

Let $F_i(N)$ be the number of establishments in the ith rank in a city of size N shown in Fig. 2A (main text). When summed over all ranks (that is, over all possible types of businesses), this must give the total number of establishments in the city, $N_f(N)$: thus,

$$\sum_{i=1}^{D_{\max}} F_i(N) = N_f(N) \tag{1}$$

Every $F_i(N)$ is a positive definite function of N, and, therefore, cannot scale faster than linearly with N ($N_f \approx \eta N$). If we re-express Eq. (1) as $\sum_i \bar{F}_i(N) = 1$ by introducing normalization, $\bar{F}_i(N) \equiv F_i(N)/N_f$, we can easily derive that individual terms are independent of N when N is growing. Any growing or diverging dependences of the $\bar{F}_i(N)$ on N cannot be cancelled against each other because of their positive definiteness, whilst any decreasing dependence vanishes for sufficiently large N. Therefore each $\bar{F}_i(N)$ or $f_i(N) \equiv F_i(N)/N$ must itself become independent of N, as verified in Fig. 2B (main text), in order to satisfy the sum rule ($N_f \approx \eta N$).

This condition holds even when we treat the discrete rank, i, as a continuous variable, x, and, correspondingly, the frequency, $F_i(N)$ and $f_i(N)$, as continuous functions, $F(x, N)$ and $f(x)$, when $D(N)$ is large enough and resolution of the categorization is in the limit of infinite (i.e., when $D_{\max} \to \infty$). Therefore it requires careful attention when focusing on the cities that have small diversity $D(N)$ and coarsed resolution r with small $D_{\max}(r)$. With this caveat, the sum rule can be well-approximated by $\int_1^{D_{max}} f(x)dx = \eta$, with $f(x) > 0$, implying that $f(x)$ should become independent of city size for sufficiently large N. The surprise in the data is that the predicted collapse into a single curve independent of city size extends down to relatively small cities (that is, up to relatively high rankings). The precocious nature of the scaling of diversity mirrors that observed in urban metrics as a function of N seems to persist down to cities with populations only in the tens of thousands of inhabitants.

2.4 The universal shape

The empirical data suggest that the universal form of this scaled rank-frequency, $f(x)$, has three distinct regimes: for small $x(< x_0$, say), it is well described by a Zipf-like power law with exponent γ, as shown in the inset of Fig. 2B; for larger $x(> x_0)$, the curve seems exponential (the approximately straight line portion in Fig. 2B); finally, as x approaches its maximally allowed value for the total number of establishment categories, $D_{\max}$ (the finite resolution of the data given the classification scheme), $f(x)$ drops off dramatically. To a very good approximation, these can be combined into a single analytic form:

$$f(x) = Ax^{-\gamma}e^{-x/x_0}\phi(x, D_{\max}) \tag{2}$$

The parameters, $\gamma \approx 0.49$ and $x_0 \approx 211$, are both independent of N, and the function, $\phi(x, D_{\max})$, parametrises the cut-off behaviour enforced by having a finite resolution; A is determined by the sum rule $f(x)$ and $f(1) \approx A \approx 0.0019$.

The general form of the cut-off function, $\phi(x, D_{\max})$, can, to a large extent, be determined by imposing three conditions:

(i) $\phi(x, D_{\max}) = 1$ when $x \ll D_{\max}$: This expresses the constraint that the cut-off is only important when x is close to $D_{\max}$.

(ii) $\phi(x, D_{\max}) \to 1$ when $D_{\max} \to \infty$: As its effect vanishes in the limit of the finest grained resolution for any value of x.

(iii) $\phi(D_{\max}, D_{\max}) = 0$: This means that the cut-off completely dominates when x reaches its maximum value determined by the finite resolution, $x = D_{\max}$.

A simple phenomenological function that satisfies all of these conditions is

$$\phi(x, D_{\max}) = e^{[1-(1-x/D_{\max})^{\xi}]} \tag{3}$$

with $\xi < 0$. Notice that $d\ln\phi(x, D_{\max})/dx = (\xi/D_{\max})(1 - x/D_{\max})^{\xi-1}$ which approaches $-\infty$ when $x \to D_{\max}$, as manifested in the data (Fig. 2B, in main text). An excellent fit to the data is obtained with $\xi \approx -1.2$. For comparison, we also show fits to the data both with and without the finite resolution cut-off function, $\phi(x, D_{\max})$. An almost equally good fit is obtained with $\xi = -1$, in which case $\phi(x, D_{\max}) = e^{-x/(D_{\max}-x)}$ and the universal distribution takes on the simple form:

$$f(x) = Ax^{-\gamma}e^{-h(x)} \tag{4}$$

where $h(x)$ is $(x/x_0)[1+x_0/(D_{\max}-x)]$ and can be further simplified as x/x_0 when $x \ll D_{\max}$.

2.5 Growing model to explain the universal shape

Ultimately we are interested in the frequency distribution $P(s|N_f,\ldots)$ that predicts the numbers of businesses of type s given N_f establishments and other characteristics of a city. This can be written as

$$P(s|N_f,\ldots) = \sum_i P(s|i,N_f\ldots)P(i|N_f,\ldots), \tag{5}$$

where $P(s|i,N_f,\ldots)$ is the probability that business type s has rank i in a city of size N_f among other characteristics. Note that $\sum_s P(s|i,N_f,\ldots) = 1$, because it is a probability and must be normalized.

The crucial finding in the main text is: 1) $P(i|N_f,\ldots)$ takes a universal form $f(i,N_f,\ldots)$, common to all cities, regardless of both their level of income and their geographical locations, as shown in Fig. 2B (main text) of the main text, and this is the reason why we deal with $P(i|N_f,\ldots)$ instead of $P(s|i,N_f,\ldots)$; 2) although $P(s|i,N_f,\ldots)$ is in general a complicated function, it is partly explained by scaling analyses in the section 3; and 3) $f(i)$ can be derived from a generalization of well known stochastic processes [3, 4]. More specifically, $F(i,N_f,\ldots)$ is the frequency of establishments with rank i, which empirically shows a universal form simply proportional to N_f. Justified by these findings we drop the $\ldots$ and write $F(i,N_f)$. This rank size distribution can be derived from a stochastic process for $f(u,N_f)$, which is the number of different business types that have appeared u times in N_f total businesses, and thus $uf(u,N_f)$ is the total number of occurrences of all businesses that appeared u times. The reason why we have to deal with $f(i)$ instead of $f(u,N_f)$ is that $f(i)$ is universal while $f(u,N_f)$ is a complicate function of both u and N_f. Recall that a fast decay (e.g. exponential) in the rank frequency distribution corresponds to slow behavior in terms of $f(u,N_f)$ and vice versa. This is well known in terms of power law distributions but needs to be generalized to our case, given the negative exponential form for the rank-size distribution at tail.

As noted in the main text the universal form of $f(i)$ takes two distinct regimes, at ranks $i < i_0$ where it behaves as a decaying power law with exponent $\gamma < 1$, and at ranks $i \gg i_0$ where it decays exponentially. In the first regime $f(i)$ is dominated by $i^{-\gamma}$, that is, $f(u) \sim u^{-1-1/\gamma}$ using the standard manipulation between rank distribution and cumulative distribution. Yule-Simon model, preferential aggregation, used not only in the economic context, but also in the ecological behavior, generates this power-law distribution with the exponent $\rho \equiv 1/\gamma$ is a function of α as $\rho = 1/(1-\alpha)$, and α is the probability of introducing a new business type where $1 \geq \alpha \geq 0$ (notation and derivation are consistent with the original

Simon paper in [3]). In this regime we can understand the distribution of business types by a process of stimulated aggregation, where common business types are likely to attract new establishments of their type (with a probability proportional to their frequency), and where new establishments are introduced with probability $\alpha = 1 - \gamma$ per introduction.

In the exponential regime, at low frequency of occurrences, the logarithmic growth in D (Fig. 1B in the main text, and Eq. (6)), are the slowest possible in terms of power laws derivable by the Yule process. Let us first derive the form of the distribution $f(u, N_f)$ that corresponds to an exponentially decaying rank-frequency.

Assuming that $f(i)$ is sufficiently described by Ae^{-i/i_0} when $i \gg i_0$, we obtain the cumulative distribution in frequency space, $P(X > u)$, as

$$P(X > u) = P(X > Ae^{-i/i_0}) \sim Bi \tag{6}$$

Changing variables so that $u = Ae^{-i/i_0}$ and thus $i = i_0 \ln(A/u)$ leads to

$$P(X > u) \sim \ln \frac{A}{u}, \tag{7}$$

which decays slowly with the value of the frequency u. Finally we obtain the probability density via differentiation with respect to u, so that

$$f(u) = \frac{B}{u} \tag{8}$$

This form of the probability density is not well defined in the continuum as integrals that include arbitrarily low frequencies result in a log-divergence in the normalization. However, as we have seen more clearly in the rank-frequency picture, there cannot be frequencies lower that $u \sim 1/N_f$ due to the discreteness of establishment numbers. In this sense the distribution (8) is well defined in our problem with a cut off.

Now, the form, Eq. (8), cannot be explained by a simple Yule process with a constant probability of introduction of new establishments. Instead we must invoke a modification where the number of new establishment types introduced is slowing down with city size. Because α is the rate of introduction of a new establishment per entry added we can write

$$\alpha(N_f) = \frac{dD(N_f)}{dN_f} = \frac{D_0}{N_f} \tag{9}$$

in the exponential region, under the assumption that $D(N_f)$ is a logarithmic function of N_f as is shown in Fig. 1 in the main text. It then can be shown [3, 4] that the exponent ρ of the

probability density corresponding to the Yule process with decreasing rates of establishment introduction is

$$\rho(N_f) = \frac{N_f\ \alpha(N_f)}{D(N_f)} \frac{1}{1-\alpha(N_f)} \tag{10}$$

The detailed derivation of Eq. (10) can be referred to the Eq. (2.34) in the original Simon's paper in [3]. In case that $N_f \to \infty$ such that $\alpha \ll 1$, and that the resolution is infinite, that is, $D_0 \ll D(N_f)$, the exponent vanishes $\rho(N_f) \to 0$. Then, the probability density $f(u, N_f)$ becomes consistent with the Eq. (8):

$$\lim_{N_f \to \infty} f(u, N_f) = \lim_{N_f \to \infty} \frac{B}{u^{1+\rho(N_f)}} \to \frac{B}{u^1}. \tag{11}$$

and the exponential form is obtained in the rank-frequency picture, as observed.

More generally we can take this slow dynamics of ρ seriously across all scales of N_f and obtain a rank size frequency that behaves like

$$F(i, N_f) = \frac{C}{i^{1/\rho(N_f)}}, \tag{12}$$

with $\rho(N_f) \simeq 1/\gamma > 1$, for small ranks, and $1/\rho \sim \infty$ for large ranks.

3 Scaling analysis and rank shifts

The universal distribution of frequency does not account for the entire developmental process of economic functionalities in cities because the stochastic model does not speak what business compositions sit in what ranks. It is indeed that the economic compositions that occupy certain ranks differ by cities, as shown in The Fig. 2A in the main text and Fig. 2 - 6 in the previous section. This dissimilarity can be studied in the historical or regional context, which is the conventional way of understanding urban economics. Here, we formalize a functional form of the proportion of components as a function of the economic size. One of the framework for such job is allometric scaling commonly used in ecology and paleontology.

Scaling exponents were calculated using ordinary least square (OLS) regression of log-transformed quantities (the number of establishments in a given sector, for example) against log-transformed population of cities. The basic urban scaling finds the total number of establishments N_f linear (the first panel of Fig. 8). Then we break the N_f into different business sectors classified by first 2 digits of NAICS. There are 19 industry sectors in NAICS each of which is scaled as the following figures: Fig. 8, 9, 10, and 11. All exponents of allometric scaling, marked in corresponding figures, are summarized in the histogram of Fig. 3 of the main text.

Because the NAICS is hierarchical which can further break into 1164 industry types, we also apply the same method to 1164 industry types. Some industry types do not have enough samples to estimate the exponents. Therefore we only include 954 industry types out of 1164 which have more than 100 samples (MSAs that have the types). The scaling exponents of all 954 types are summarized in Fig. 12. As labeled in the figure, the conclusion is consistent with the Fig. 3 in the main text in that primary industries shift out while higher industries shift in as a city is larger.

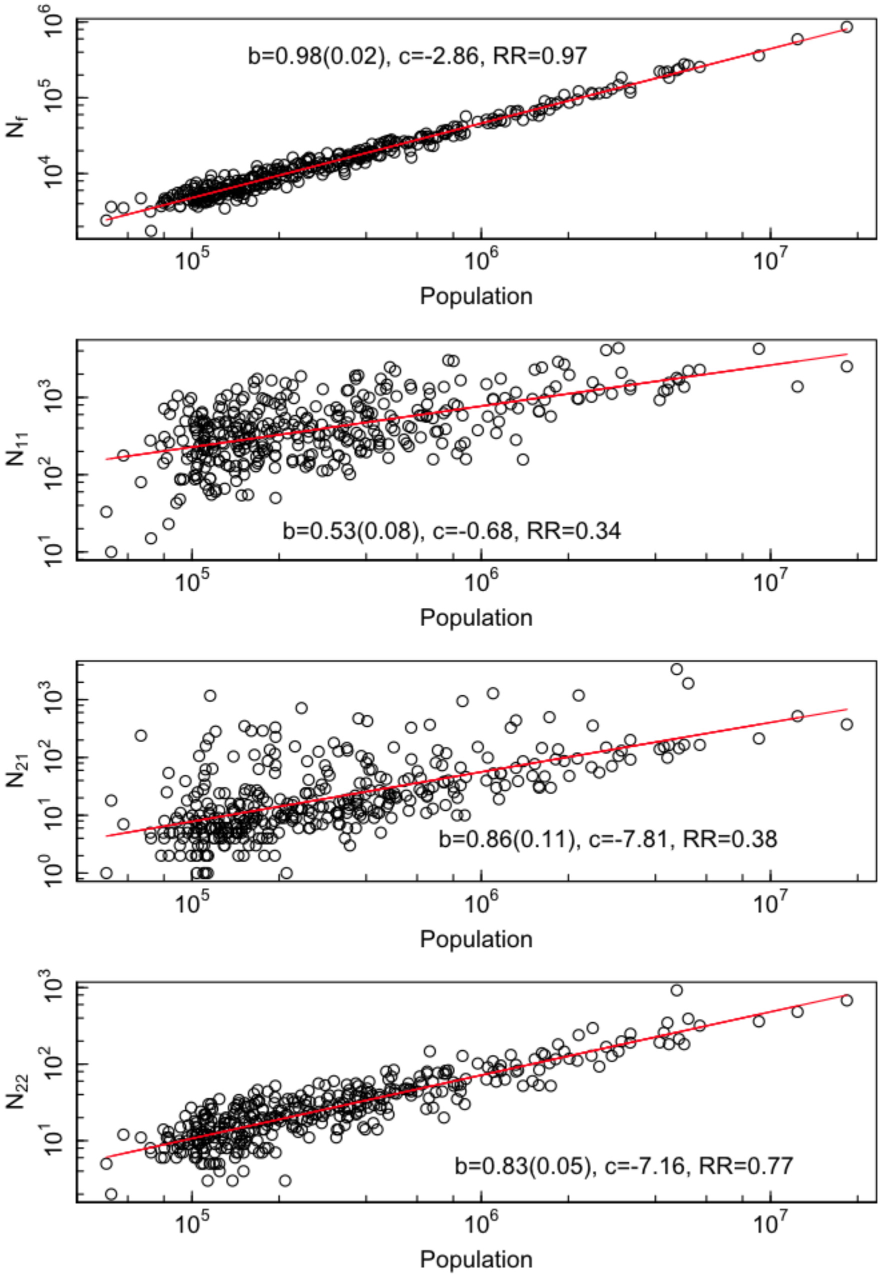

Figure 8: Scaling of the number of establishments of in cities for each industrial types. From the top, is, total, agriculture, forestry, fishing and hunting (11), mining (21), utilities (22),

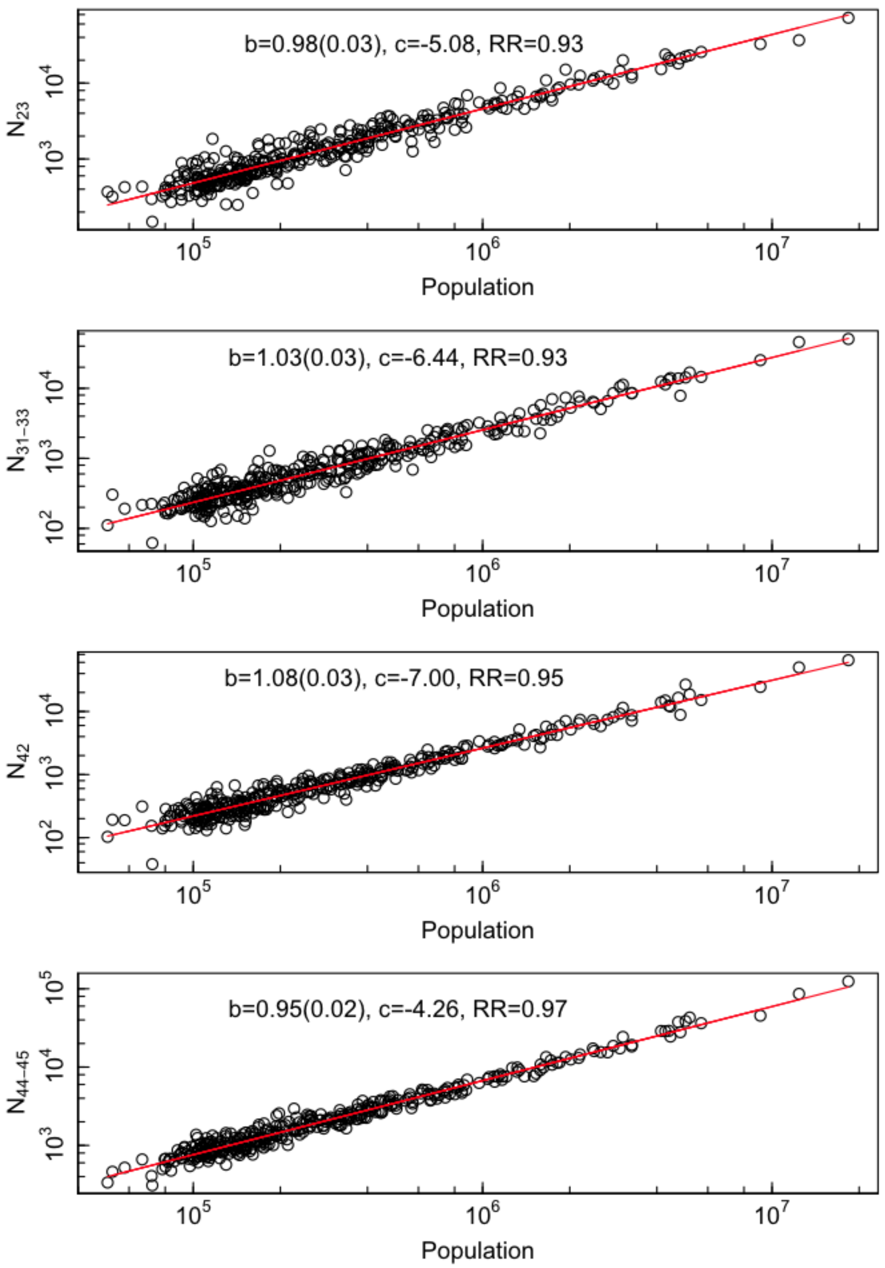

Figure 9: Scaling of the number of establishments in cities for each industrial types. From the top, is, construction (23), manufacturing (31-33), wholesale trade (42), retail trade (44-45).

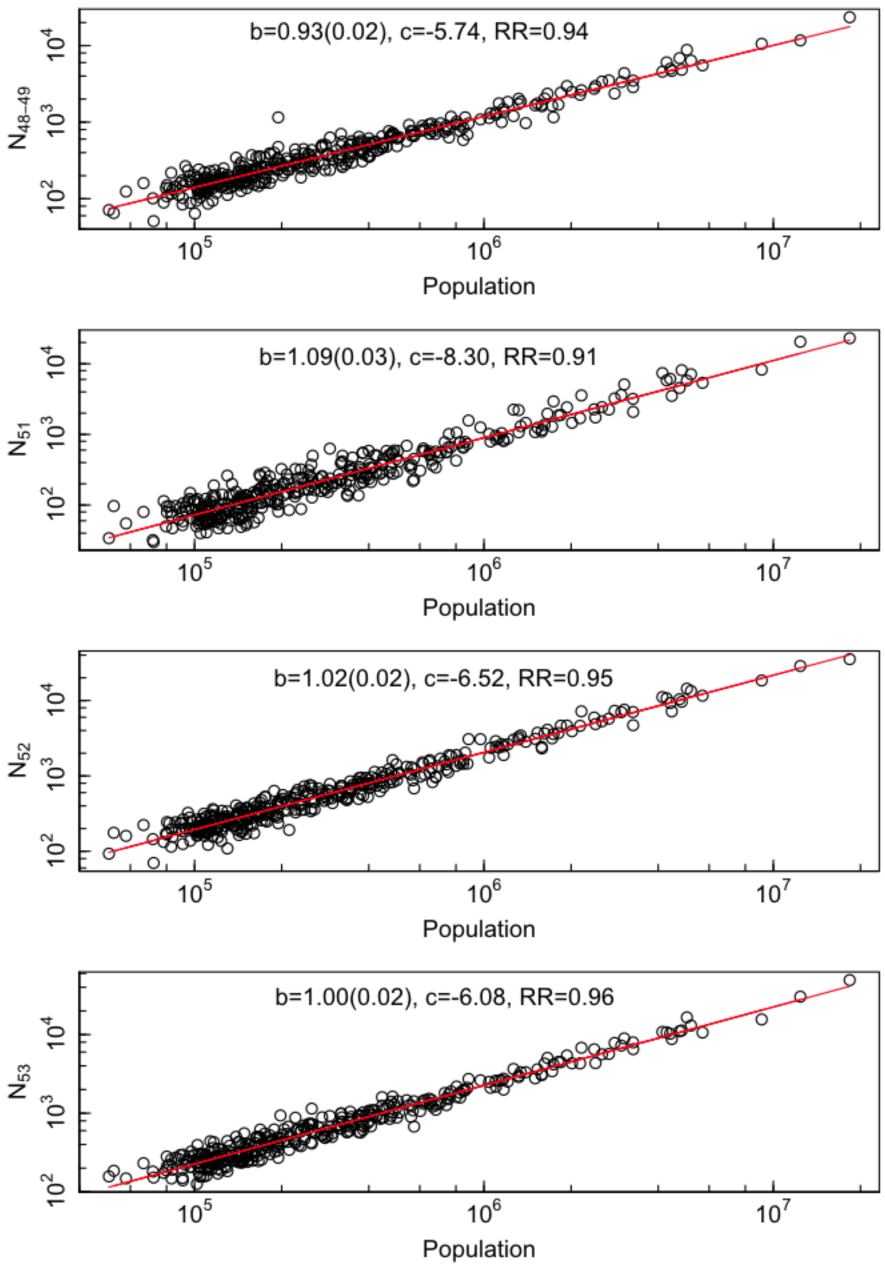

Figure 10: Scaling of the number of establishments in cities for each following industrial type. From the top, is, transportation and warehousing (48-49), information (51), finance and insurance (52), and real estate rental and leasing (53).

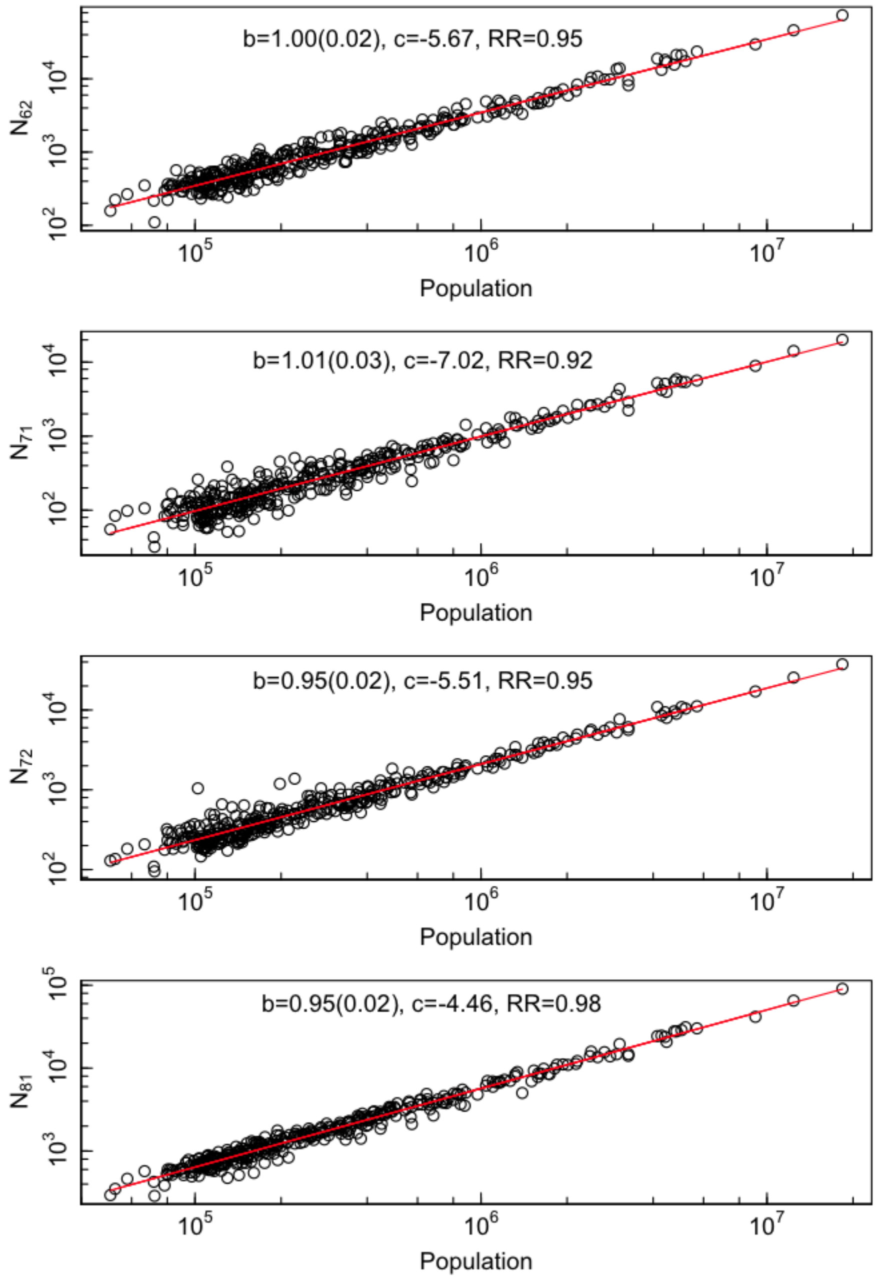

Figure 11: Scaling of the number of establishments in a city for each following industrial type. From the top, is, health care and social assistance (62), arts, entertainment, and recreation (71), accommodation and food services (72), and other services except public administration (81).

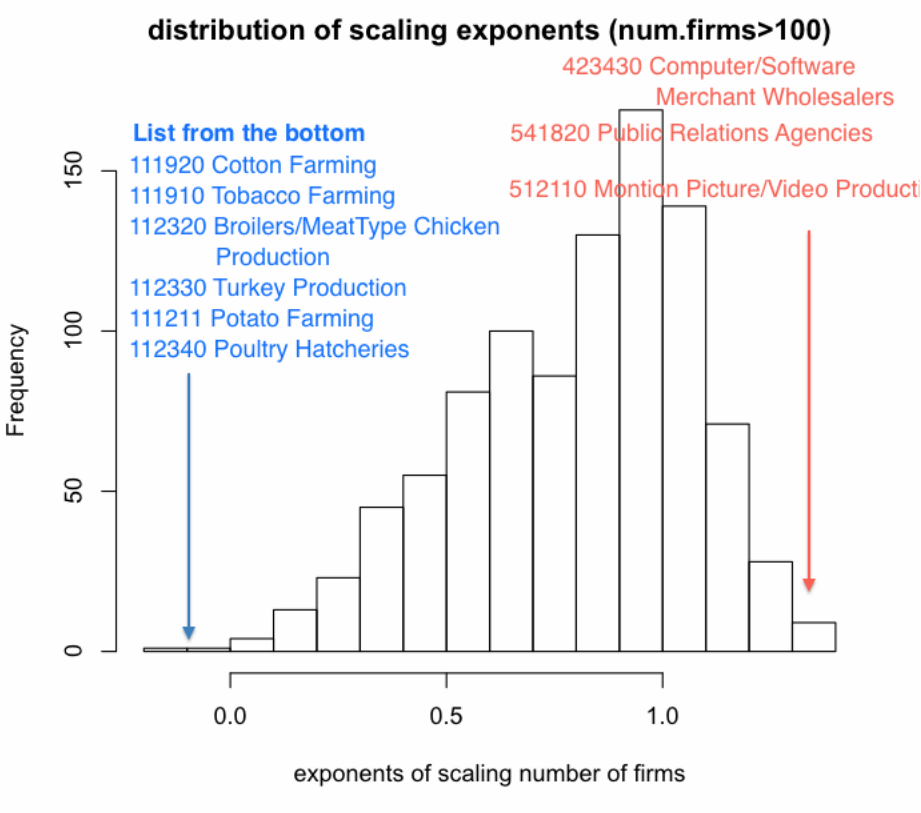

Figure 12: Histogram of scaling exponents at 6 digit level of NAICS. Only more than hundred data points are scaled and contribute to the histogram.

References

[1] Davis SJ, Haltiwanger JC, Schuh S (1998) *Job Creation and Destruction* (The MIT Press)

[2] North American Industry Classification System: http://www.census.gov/eos/www/naics/

[3] Simon HA (1955) *Biometrika* 42:425.

[4] Zanette DH and Montemurro MA (2005) *J. Quantitative Linguistics* 12:29-40 (2005).
